# Precision Medicine: What Do We Expect in the Scope of Basic Biomedical Sciences?

**DOI:** 10.1016/j.gpb.2016.02.001

**Published:** 2016-02-12

**Authors:** Jun Yu

**Affiliations:** CAS Key Laboratory of Genome Sciences and Information, Beijing Institute of Genomics, Chinese Academy of Sciences, Beijing 100101, China

The era of Precision Medicine (PM) has entered its execution phase where acquisition of human genome variation data is already leading the charge [1], [2]. PM has come a long way to this phase from the Human Genome Project started some 25 years ago [3], [4]; it marks the beginning of disease-centric research in the field of biomedical sciences. Other than delivering high-coverage human genomes by millions—of course tailored to patients and common diseases—in the next decade or so, what else do we expect to have and what should we be doing to make the best out of PM projects beyond the current expectation of genetics?

Believe or not, the PM bandwagon is still largely bannered with slogans promoting large-scale, data-driven (or discovery-driven) approaches, such as in-depth discovery of cancer-causing mutations, drug targets, and biomarkers, and may be coupled with hypotheses to balance the currently-debated environment-centric *vs.* mutation-centric arguments. Nevertheless, we are still in time to speculate how to grasp disease-centric PM tasks, largely focusing on various common diseases, which are clear in priority and complex in nature, including but not limited to cancers, as well as cardiovascular, infectious, metabolic, and neurological diseases or disorders. Other than disease-oriented objectives, we should think more and deeper about other useful data and well-thought tasks in addition to genome sequences, as well as new synthesis as to how to acquire novel data and to interpret them with wisdom [5], [6], [7].

First of all, once we have an enormous amount of high-quality sequences, understanding the human population structures and defining haplotypes within and between populations, as well as their disease relevance, are of essence. As the nature of this particular venture is largely informational, population-based sequence variation databases [8] are long-awaited, since the raw data accumulation may exceed the current computation and storage capacities. An integrated database hosts all sequence variations and functional annotation is highly desirable. In addition, mutation biases can also be further used to define function-selected sequence elements beyond protein-coding sequences [9], [10]. For instance, when human genes are partitioned into house-keeping and tissue-specific genes, the mutation rate of tissue-specific genes appear 33% higher than that of house-keeping genes [11], [12]. This observation suggests that the germline associated genes and chromosome organization hold keys to a variable mutation rate. Another example is the fact that the size of universal introns (size-invariable within lineages) are found—based on population data—to be functionally selected toward size optima, albeit lineage-associated [9], [13]. After all, mutations are neither created randomly nor equally in an operational sense for overall DNA sequences and their carriers—chromosomes, let alone selections that are largely attributable to function or phenotype and poorly defined in structural terms of genes and intergenic sequences [14].

Second, studying cellular gene expression and regulation in precision presents another challenge. As a compartmental unit of life, cellular heterogeneity provides functional diversity, even when spoken about asexually divided bacteria. Although transcriptomics is well-respected as a critical paradigm for gene expression study tailored to cells [15], not yet has a single standard human transcriptome been produced, claimed, validated, and hosted in an authorized database. The difficulties are enormous at present time [16]. We have not yet been able to sequence RNA directly in a resolution of copies per cell and to define chemically-modified RNA sequences quantitatively at single-molecule resolution [17], [18], [19]. We have not yet been able to separate cellular RNAs into appropriate classes, ribosomal *vs.* total, messenger *vs*. non-coding, small *vs*. large, *etc*. All these call upon a genome-wide large-scale project world-wide: the Human Transcriptomes Project. The Human Transcriptomes Project will certainly come after the sequencing effort in the early phase of the PM project, or being a sequel of it or maybe even sooner, as the current sequencing capacity and tasks will have to be redirected after the genome sequencing effort reaches a peak.

Third, defining DNA structural elements and gene organization as landmarks of chromosomes is undoubtedly a major endeavor. The ENCODE project has been paving ways for thorough definition of operational DNA elements for each cell type and tissue (http://www.genome.gov/encode/). There are quite a few unanswered questions along the line. For instance, most human genes are organized into clusters but circadian-regulated genes are cluster-avoiding. How are they synchronized in expression and organized into chromosome territories [14]? How transcript-rich cells, such as testis, brain, and stem cells, are organized to express most of their genes [20], [21]? How chromosomes are organized, inherited, and regulated in step-wise changes precisely in germline cells to ensure body development from a zygote? Obviously, a human chromosome-based gene organization map becomes important, which may include experimental data and information, such as sites of chemical modifications, gene clustering and regulation (such as antisense transcription-based regulation), nucleosome occupancy (density *vs.* expression levels), non-transcribed regulatory elements, and organizer-anchorage sequences. We still have a long way toward three-dimensional modeling of the human chromosomes in a dynamic way for development and differentiation.

Fourth, other than informational and operational (structural and interactive functions) systems, rules and nature of various homeostatic processes are also critical and unique, including generation and control of energy, material, and signal transduction. The leptin-adipocyte signal control system represents an excellent example; the discovery of *obese* (*ob*) gene and its mutation in mouse has not yet led to an ultimate cure for obesity [22]. In this particular regime, cellular processes involved in cross-physiological systems are to be deciphered and large PM projects to categorize components and metabolites in circulating and excreting body fluids are to be expected. To measure everything in precision, novel assays and instrumentation are both essential.

Fifth, some PM projects have to go longitudinal as life is after all governed by time. In the dimension of time, we have so many mysteries to be solved; in addition to normal development and aging, there are more than enough symptoms to be reduced and maybe even cured, including menopause syndrome, Alzheimer’s disease, osteoporosis, osteoarthritis, diabetes, just to name a few. In this regime, plasticity or cellular responsiveness to stress signals and materials comes to the center stage, and the degree and timing are both to be measured in precision. Whether the relevant PM projects are named exposomes, stressomes, or plastisomes may not be important but time-lapse records and measures are the keys. The connectome projects for neurology of several model organisms together with the Human Connectome Project have been pioneering on cognitive plasticity (http://www.humanconnectomeproject.org/). Similar projects on the lymphatic system have also come to their time.

It is clear that the stratification of biology into distinct systems is equally important to that of diseases, as we are increasingly capable of exploiting new territories of research fields [23], [24]. The fields of genetics, epigenetics, and environment have not provided enough conceptual freedom to allow precise description of genotype–phenotype relationship, where complexity, phenotypic plasticity, and cellular heterogeneity are conceptions frequently used. On the one hand, multi-track biology takes a divide-and-conquer approach to define useful data for understanding disease mechanisms at molecular levels. On the other hand, multi-track biology also takes a systematic approach to integrate data into the information commons that can be synthesized into knowledge on physiological systems as well as diseases, the pathological states of the physiological systems. The medicine side of the PM projects is also disease-centric, and their success largely depends on the organization of cohorts; we also expect the data from this side merges into the same information commons, where mechanisms of diseases are deciphered and strategies are designed to make humans healthier more than ever.

## Competing interests

The author declared that there is no competing interest.
